# Post‐Extubation Recall of Thirst During Mechanical Ventilation Among Intensive Care Unit Patients: A Conventional Content Analysis

**DOI:** 10.1002/nop2.70912

**Published:** 2026-10-09

**Authors:** Alireza Sadeghi, Amir Mohamad Nazari, Alun C. Jackson, Elham Khedmati, Fatemeh Bahramnezhad

**Affiliations:** ^1^ Student Research Committee, School of Nursing and Midwifery Tehran University of Medical Sciences Tehran Iran; ^2^ Medical‐Surgical Nursing, Student Research Committee, School of Nursing and Midwifery Shahid Beheshti University of Medical Sciences Tehran Iran; ^3^ Australian Centre for Heart Health Melbourne Australia; ^4^ Department of Allied Medical Sciences Tehran University of Medical Sciences Tehran Iran; ^5^ School of Nursing and Midwifery, Nursing and Midwifery Care Research Center Tehran University of Medical Sciences Tehran Iran

**Keywords:** critical care, intensive care units, qualitative research, thirst

## Abstract

**Background and Aim:**

In ICU patients, particularly after extubation, thirst is a common and troubling symptom. Although earlier research has focused mainly on quantifying thirst severity and its clinical correlates, less is known about the subjective experiences of patients, including their emotional responses, sense of helplessness, help‐seeking actions, and the challenges they face in obtaining relief within the ICU. Therefore, this study aimed to examine how post‐extubation ICU patients experience thirst and the strategies they use to manage or alleviate it.

**Methods and Materials:**

Between January and August 2024, this qualitative research was carried out in Tehran, Iran, using conventional content analysis. Participants were recruited through purposive sampling, and data were collected via semi‐structured interviews conducted in Persian with 20 patients admitted to the intensive care unit (ICU) following extubation. Interviews proceeded until data saturation occurred, and even though they were held after extubation, the thirst experiences participants reported mostly pertained to the time of mechanical ventilation and intubation. The Consolidated Criteria for Reporting Qualitative Research (COREQ) guideline was followed in this study.

**Results:**

Three main categories were identified in this study: ‘Unrelieved Thirst’ (Hope despite Disappointment and Cognitive and Emotional Burden of Thirst), ‘Seeking Relief’ (Spiritual Coping and Endurance and Non‐Verbal Pleading for Water), and ‘Emotional Collapse and Withdrawal’ (Resigned Withdrawal and Emotional Isolation and Perceived Neglect). Participants experienced persistent thoughts about water, employed spiritual coping strategies, used gestures without words to express thirst, and eventually emotionally withdrew when their needs were persistently ignored.

**Conclusion:**

The study revealed that post‐extubation thirst is experienced as a deeply upsetting phenomenon affecting physical, psychological, and existential levels, where patients face physical discomfort, emotional distress, challenges in communication, and a sense of being overlooked in the ICU.

**Implications for the Profession and/or Patient Care:**

The findings highlight the urgent necessity for systematic thirst assessment, improved non‐verbal communication techniques, and compassionate, patient‐focused nursing care to reduce thirst‐related distress. Healthcare providers must be trained to identify both verbal and non‐verbal thirst signals and to incorporate patients' cultural and spiritual resources into supportive care.

**Impact:**

The research delivers a comprehensive understanding of the multifaceted nature of thirst in ICU patients, demonstrating that unresolved thirst may result in emotional collapse and withdrawal. It highlights the significance of managing thirst as an essential human requirement in critical care.

**Reporting Method:**

The study followed the Consolidated Criteria for Reporting Qualitative Research (COREQ) guideline.

**Patient or Public Contribution:**

No patient or public contribution.

## 1 Introduction

Patients admitted to the intensive care unit (ICU) often experience thirst, which is a common complaint and significant distress, influenced by factors such as mechanical ventilation, endotracheal intubation, negative fluid balance, and the use of diuretics and analgesics, though additional clinical and treatment‐related elements may also play a role (Stotts et al. 2015). Although the need to drink water is vital for fluid balance and physiological stability, in critically ill patients, thirst goes beyond a mere physiological signal and may cause significant discomfort and distress. Although thirst is increasingly acknowledged as a clinically important symptom, there is still a significant lack of understanding regarding the patient's subjective experience of thirst during ICU admission (Yuan et al. 2025). Understanding thirst in ICU patients necessitates attention to its biological regulatory processes as well as its subjective emotional meanings (Kjeldsen et al. 2018). These insights highlight the psychological facet of thirst, but the way patients experience and interpret thirst in the ICU environment has not been thoroughly explored.

Grounded in the Theory of Unpleasant Symptoms (TOUS) (Lenz et al. 1997), this study utilizes a multidimensional framework to understand how symptoms are influenced by physiological, psychological, and situational factors, resulting in diverse performance outcomes. Although the original theory did not explicitly illustrate thirst, later studies (Best et al. 2026; Oyama et al. 2025) have used this framework to investigate thirst as a complex symptom in patient groups, including the critically ill. This theoretical perspective provides a thorough method for analysing the multifaceted nature of thirst in critically ill individuals. In this study, the TOUS served solely as a sensitizing background framework and an interpretive lens in the Discussion; it was not employed as a deductive coding framework, nor were any a priori codes or categories derived from it.

Patients undergoing prolonged ICU care and mechanical ventilation often report thirst as one of the most upsetting elements of their experience, usually coupled with feelings of fear, anxiety, and helplessness. Even though intubated patients cannot express their thirst in real time due to their inability to speak, their retrospective reports after extubation indicate that thirst may be especially intense during intubation, when they cannot drink freely or effectively communicate their needs (Xie et al. 2025). Although these findings exist, there is little evidence on how ICU patients perceive, interpret, and manage thirst during their treatment. Because this study aimed to investigate patients' subjective and retrospective experiences after extubation, a qualitative approach was specifically selected, as it is ideal for areas with limited existing knowledge and allows the researcher to richly describe participants' experiences without applying predefined theoretical categories. Therefore, this study aimed to examine how post‐extubation ICU patients experience thirst and the strategies they use to manage or alleviate it.

## 2 Methods

### 2.1 Study Design and Participants

In this study, data were gathered from January to August 2024 across three intensive care units in teaching hospitals (Imam Khomeini Hospital Complex, Mahdi Clinic, and Valiasr Hospital) associated with Tehran University of Medical Sciences, Tehran, Iran. This study used a qualitative design with conventional content analysis, selected due to limited existing knowledge about ICU patients' thirst experiences, and because this method effectively describes participants' experiences without applying preconceived theories. Conventional content analysis involves systematically describing and categorizing participants' accounts. For this study, analysis covered both manifest and latent content, starting with manifest content, which provided the basis for coding and categorization. The process of abstraction also involved careful consideration of latent content, including psychological, existential, and spiritual meanings indirectly expressed or embedded in participants' accounts, with interpretations rooted in the data and not based on pre‐existing theories. Accordingly, while the analysis was inductive and grounded in participants' narratives, the depth of interpretation reflected the exploratory nature of the study.

Adhering to the Consolidated Criteria for Reporting Qualitative Research (COREQ) guideline, this study ensured transparent reporting of the research team, reflexivity, sampling, data collection, analysis, and presentation of the findings. Participants were recruited using maximum variation purposive sampling to gather rich information about the thirst experience, seeking diversity in characteristics that might affect thirst in the ICU such as age, sex, ventilation duration and ICU admission reason. The clinical heterogeneity of the sample was purposeful and consistent with the maximum variation sampling strategy, as we intentionally included patients with varied diagnoses (sepsis, pneumonia, cancer, liver failure, postoperative conditions, myasthenia gravis, and suicide attempts) to encompass a wide spectrum of thirst experiences in diverse clinical settings.

This method enabled us to recognize shared patterns of thirst experience beyond individual diagnoses, whereas also examining how various clinical conditions could influence the experience. The first author identified eligible patients daily by reviewing medical records and consulting with bedside nurses and physicians. After extubation, patients meeting the inclusion criteria were approached and informed about the study's purpose. Those who consented were enrolled consecutively. In order to achieve maximum variation, we systematically monitored the sample composition during recruitment and persisted in sampling until diversity was present across age, sex, ventilation length, and ICU admission reasons. We prioritized eligible patients with a particular characteristic (like prolonged ventilation or a specific admission diagnosis) when that characteristic was underrepresented. Out of 28 eligible patients approached, 20 consented to participate while 8 declined due to fatigue, reluctance to discuss the experience, or early transfer from the ICU. The final sample of 20 participants was chosen based on their willingness to join, effective communication skills, and the variety they added to the overall sample. The sample size was set based on data saturation, reached after conducting 20 interviews.

Patients were considered eligible if they were alert, able to communicate in Persian, had a RASS score within −1 to +1, exhibited no delirium on the CAM‐ICU, and were contacted 5 h following extubation. Although a RASS score between −1 and +1 indicated alertness, readiness for the interview was evaluated more thoroughly by considering the patient's clinical stability, communication ability, willingness to engage, and tolerance for the interview. Patients were excluded if they had a tracheostomy, underwent maxillofacial or oral surgery, had head and neck cancer, required sedation, suffered from alcohol or drug abuse, or were deaf.

### 2.2 Data Collection

All interviews were conducted and recorded by the corresponding author, a female PhD in nursing with a decade of clinical experience in intensive care. She had no previous relationship with the participants, which was clarified to them prior to obtaining informed consent. Participants received information about the researcher's professional background, the study's objectives, and the rationale behind the research prior to consenting.

The reported 40–60 min duration covered the entire interview session, including any needed breaks, with each interview conducted flexibly based on the participant's clinical condition and tolerance. Interviews proceeded until data saturation was reached. In total, 20 semi‐structured interviews were carried out, with most initial codes arising from the first 12 interviews. The next five interviews (13–17) focused on confirming and refining the emerging categories, with the final three interviews (18–20) uncovering no new codes, which indicates data saturation was achieved. Saturation was evaluated by the first author and validated through consultation with co‐authors, who examined the coding framework and agreed that no additional codes or categories were arising.

Data collection and analysis occurred simultaneously; after each interview which was recorded by audio recorder, the transcript was coded, and the emerging codes and categories guided the focus of the next interviews. This iterative approach enabled us to refine probing questions and delve deeper into emerging concepts in subsequent interviews. As the subcategory ‘Spiritual Coping and Endurance’ surfaced in initial interviews, following interviews featured more specific questions regarding spiritual and religious coping strategies. Every session started with general guiding questions such as: ‘Can you describe your experience of thirst during your stay in the ICU?’ and ‘How did thirst make you feel physically and emotionally?’ When suitable, participants were additionally asked: ‘What did you do, or what did you want to do, to relieve your thirst?’ Afterwards, probing questions were asked for clarification and further detail. Although interviews occurred post‐extubation, the thirst experiences shared by participants mainly related to the time they were mechanically ventilated and intubated. Participants were specifically requested to remember and describe their thirst experiences while they were intubated and on mechanical ventilation.

To guarantee thorough data, participants were ultimately asked if any aspect of their experience had been overlooked, with interviews conducted about 5 h post‐extubation. This timing was chosen to obtain fresh and detailed memories of the intubation period while reducing recall bias and to confirm that patients had reached adequate clinical stability and recovered from the immediate effects of intubation. However, the specific interview time was tailored and decided based on the patient's readiness and preference in coordination with the bedside nurse or physician. If participants experienced throat pain, hoarseness, or fatigue, interviews were paused, shortened, or rescheduled, and they were allowed to end the interview whenever they wished.

To address the inherent difficulties of the ICU setting and the immediate post‐extubation phase, particular strategies were implemented, with interview timing carefully coordinated alongside bedside nursing staff to select times of reduced clinical activity and noise, like during non‐peak hours or between essential care procedures. To enhance patient comfort and focus, these quieter windows were given priority. Additionally, clear communication protocols with nurses were set up to guarantee patients were both clinically stable and mentally ready for the interview, reducing disruptions and potential fatigue.

### 2.3 Data Analysis

While the Theory of Unpleasant Symptoms (TOUS) provided a sensitizing framework to understand the multidimensional aspects of thirst, it was not used deductively for coding. No pre‐established codes, subcategories, or categories were derived from TOUS; rather, all codes and categories emerged inductively from the participants' descriptions. TOUS was employed only in the Introduction to contextualize the study and in the discussion to interpret results after completing the inductive analysis.

The data analysis followed the traditional content analysis method outlined by Graneheim and Lundman (2004). Data were manually analysed without employing qualitative data analysis software. Transcripts were organized and managed using Microsoft Word tables, whereas codes, subcategories, and categories were monitored through manual colour‐coding and repeated comparison across transcripts. Immediately following each interview, the verbatim transcription was done and reviewed several times to gain a comprehensive understanding of the content, after which the text was segmented into meaning units related to the participants' thirst experiences. These meaning units were condensed without losing their core meaning and assigned initial codes. The codes were continuously compared across interviews for similarities and differences and grouped into subcategories and broader categories. Data collection and analysis occurred simultaneously, enabling emerging insights to guide subsequent interviews.

According to Graneheim and Lundman (2004), both manifest and latent content were analysed; manifest content referred to participants' direct descriptions of thirst and related experiences, whereas latent content concerned the deeper psychological, existential, and spiritual meanings implied or embedded in their accounts. The initial coding focused primarily on manifest content, while latent meanings appeared during abstraction and category development. All latent interpretations were examined by the research team and cross‐checked with the data to ensure they were grounded in participants' accounts.

The primary coding of all 20 transcripts was carried out by the corresponding author, while a second coder experienced in qualitative research independently coded five transcripts (25%). These transcripts were deliberately chosen to reflect maximum variation in age, sex, ventilation duration, and ICU admission reason. The corresponding author and the second coder reviewed their codes, subcategories, and categories, addressing disagreements via structured team discussions; if they failed to reach consensus, a senior qualitative methodology expert was consulted, and the final decision was collaboratively determined. Moreover, throughout the analysis, the other authors repeatedly reviewed all codes, subcategories, and categories, with all analytic decisions and resolutions documented in an audit trail.

### 2.4 Translation Process

The corresponding author, a native Persian speaker holding a PhD in nursing and with 10 years of clinical intensive care experience, translated all participant quotations and qualitative findings from Persian to English. She is fluent in English and well acquainted with the cultural and clinical context of the intensive care unit. An independent bilingual translator, fluent in Persian and English and uninvolved in the original translation or analysis, back‐translated all translated quotations into Persian to verify semantic and conceptual equivalence. These back‐translations were subsequently compared with the original Persian transcripts by the corresponding author and a bilingual co‐author. Discrepancies in meaning, emotional tone, or cultural nuance were thoroughly discussed and resolved until consensus was reached, and the research team examined the final English translations in team meetings to confirm fidelity to the participants' accounts.

### 2.5 Rigour

The purpose of rigour is to represent the participant's experience correctly (Ahmed 2024). Several terms describe the process leading to the trustworthiness of qualitative research data. Lincoln and Guba (1982) defined the terms ‘Credibility’, ‘Dependability’, ‘Transferability’, and ‘Confirmability’ to describe practical methods for achieving trustworthiness in qualitative research (Lincoln and Guba 1985).

Credibility was strengthened by conducting in‐depth semi‐structured interviews, engaging with the data for an extended period, performing member checking, peer debriefing, and investigator triangulation. For member checking, the transcribed interviews along with the initial codes and categories were sent back to 8 participants who consented to review them. The participants were asked to confirm the accuracy of the transcripts in representing their experiences and to see if the codes and categories matched their accounts, with all 8 confirming that both transcripts and categories accurately represented their experiences. Two participants suggested slight wording clarifications for certain categories, which were subsequently improved. The participant feedback did not call for any major alterations to the categories.

The primary coding of all transcripts was carried out by the corresponding author. To minimize individual bias and enhance data interpretation, the emerging codes, subcategories, and categories were repeatedly reviewed and discussed with other research team members. Investigator triangulation involved independent coding of five transcripts (25%) by a second coder experienced in qualitative research, followed by comparison of codes, subcategories, and categories between the corresponding author and the second coder, with a third senior qualitative researcher resolving disagreements when consensus was not achieved, ongoing review of the coding framework, subcategories, and categories by the broader research team, and keeping an audit trail of all analytic choices. Discrepancies in interpretation were resolved by team discussion, and when agreement was not possible, the third senior researcher made the final adjudication.

Dependability was ensured by keeping a clear and systematic record of the research process, documenting decisions made during data collection, coding, and category development to maintain a consistent and traceable analytic process. Confirmability was reinforced by documenting an audit trail of analytic decisions and engaging several qualitative research experts, such as two faculty from the School of Nursing and Midwifery and one from the School of Social Sciences, to review the coding process and findings. Transferability was improved by including direct quotations and offering a thorough description of the study context, participants, sampling, data collection, and analysis process, while reflexivity was considered by recognizing the researcher's role as an ICU nurse, a PhD in nursing, and a university faculty member. With a decade of clinical experience in intensive care units, the corresponding author maintained a reflexive journal to note her assumptions, preconceptions, and emotional responses during data collection and analysis. She regularly held peer debriefing sessions with co‐authors to examine the potential impact of her clinical background on the results. Throughout the interviews, she used open‐ended questions, refrained from leading questions that might confirm her assumptions, and motivated participants to describe their experiences personally, also investigating negative or contradictory examples.

### 2.6 Ethical Considerations

This study complied with ethical standards according to the Helsinki Declaration and was approved by the Ethics Committee of Tehran University of Medical Sciences (No: IR.TUMS.MEDICINE.REC.1400.405); to address ethical concerns, the researchers introduced themselves, clarified the research goals, and requested participants to sign informed consent forms. Prior to every interview, participants gave consent for voice recording and were assured their names would be kept confidential in any published findings, with only anonymized data reported; they were also promised that audio files would be erased after the final report. Upon request, participants were provided with a copy of their interview transcript for review and informed about the overall study results, with the guarantee that they could leave the interview whenever they wished.

Decisional capacity for informed consent was evaluated independently of clinical stability and interview tolerance. The researcher explained the study's aims, methods, risks, and voluntary participation to participants before consent, then asked them to describe these in their own words. Only patients who exhibited understanding, appreciation, reasoning, and the capacity to express a choice were enrolled. The interview readiness was evaluated individually by considering clinical stability, communication ability, willingness to participate, and interview tolerance.

## 3 Results

This study involved interviewing 20 patients hospitalized in the intensive care unit after extubation; according to the CAM‐ICU assessment, none exhibited delirium, and their RASS scores varied between −1 and +1. The demographic characteristics of the participants are detailed in Table 1.

**TABLE 1:** The demographic characteristics of the patients (*n* = 20).

ID	Age	Gender	Marital status	ICU LOS (day)	Diagnosis	MV duration (day)	Intubation duration (day)	Sedation	Fluid restriction/NPO	Diuretics	RASS	CAM‐ICU
1	29	Female	Single	18	Acidosis Metabolic	14	14	Yes (midazolam)	Yes	Yes	−1	Negative
2	42	Male	Single	10	Haemorrhage	7	7	Yes (propofol)	Yes	No	−1	Negative
3	37	Female	Married	9	Suicide	6	6	Yes (midazolam)	Yes	Yes	−1	Negative
4	39	Male	Married	6	Chest pain after surgery	3	3	Yes (fentanyl)	Yes	No	0	Negative
5	45	Male	Single	3	Abdominal surgery	2	2	Yes (propofol)	Yes	No	0	Negative
6	38	Female	Married	9	Shortness of breath due to liver failure	5	5	Yes (midazolam)	Yes	Yes	0	Negative
7	65	Male	Married	4	Pneumonia	3	3	No	Yes	No	+1	Negative
8	51	Female	Married	2	Uterine and ovarian cancer	1	1	Yes (propofol)	Yes	Yes	0	Negative
9	55	Female	Single	4	Sepsis	3	3	Yes (midazolam)	Yes	Yes	0	Negative
10	39	Male	Single	8	Bone marrow cancer	5	5	Yes (fentanyl)	Yes	No	0	Negative
11	45	Female	Married	2	Abdominal Surgery	1	1	Yes (propofol)	Yes	No	0	Negative
12	32	Female	Single	4	Sepsis	3	3	Yes (midazolam)	Yes	Yes	0	Negative
13	55	Female	Single	7	Pneumonia	5	5	No	Yes	No	+1	Negative
14	39	Male	Married	7	Myasthenia gravis	6	6	Yes (propofol)	Yes	No	−1	Negative
15	50	Male	Married	6	Sepsis	4	4	Yes (midazolam)	Yes	Yes	0	Negative
16	48	Female	Single	3	Sepsis	2	2	Yes (fentanyl)	Yes	No	0	Negative
17	61	Female	Married	4	Pneumonia	3	3	No	Yes	No	+1	Negative
18	41	Female	Single	5	Uterine and ovarian cancer	4	4	Yes (propofol)	Yes	Yes	0	Negative
19	38	Male	Single	21	Suicide	18	18	Yes (midazolam)	Yes	Yes	−1	Negative
20	56	Female	Single	11	Uterine Cancer	8	8	Yes (propofol)	Yes	No	−1	Negative

Abbreviations: ICU, intensive care unit; LOS, length of stay; MV, mechanical ventilation; NPO, nil per os (nothing by mouth).

A total of 501 open codes were extracted from 20 interviews in this study, then carefully reviewed and merged based on their content and semantic similarities to produce a reduced number of codes. The refined codes were subsequently grouped into six subcategories based on their conceptual connections, and then, through continuous comparative analysis and abstraction, these six subcategories were merged into three primary categories: ‘Unrelieved Thirst’, ‘Seeking Relief’, and ‘Emotional Collapse and Withdrawal’. Some variations were observed: patients undergoing prolonged ventilation (e.g., sepsis, suicide attempts) reported stronger thirst and greater frustration, whereas postoperative patients experienced shorter but still troubling thirst episodes. The extracted code tree is presented in Table 2, while its graphical representation is displayed in Figure 1.

**TABLE 2:** Categories, subcategories, meaning units, and supporting quote.

Category	Subcategory	Initial codes	Condensed meaning unit	Supporting quote
Unrelieved Thirst	Hope Despite Disappointment	«This cannot last forever»; «Someone will give me water»; «God will not leave me alone»	Expressions of persistent optimism despite repeated failure to receive water, characterized by belief in eventual relief and temporary nature of suffering	‘I kept telling myself that this cannot last forever. One day, someone will understand and give me water. That thought was the only thing that stopped me from giving up completely’ (Participant 6)
	Cognitive and Emotional Burden of Thirst	«Water was all I could think about»; «My mind was stuck in a loop»; «Maybe this thirst is good for me»; «Afraid to sleep»	Continuous mental preoccupation with water combined with forced endurance of deprivation; intrusive thinking patterns and emotional distress	‘Water was all I could think about. Every second, I imagined drinking a glass of cold water. It was like my mind was stuck in a loop, and I couldn't get out of it’ (Participant 14)
Seeking Relief	Spiritual Coping and Endurance	«I asked God for help»; «I remembered Imam Hussein»; «I felt closer to him»; «God was with me»	Turning to God or religious figures through prayer and supplication; finding meaning and comfort in spiritual narratives such as the story of Imam Hussein (AS)	‘Every night, I prayed to God and asked Imam Hussein to give me strength. I remembered how thirsty he was in Karbala, and I felt closer to him in my suffering’ (Participant 16)
	Non‐Verbal Pleading for Water	«I begged with my eyebrows»; «I moved my hands»; «I pointed to my mouth»; «I kept tapping on the bed»	Non‐verbal communication attempts through body language, eye movements, facial expressions, and hand gestures to convey thirst to healthcare providers	‘I tried so hard to show them I needed water. I moved my hands, I pointed to my mouth, I made eye contact and begged with my eyes. But they didn't seem to understand’ (Participant 4)
Emotional Collapse and Withdrawal	Resigned Withdrawal	«I stopped asking»; «I gave up»; «Nobody listened»; «I waited for it to end»	Gradual withdrawal from help‐seeking efforts due to repeated failures and feelings of being misunderstood, leading to resignation and despair	‘After so many tries, I just gave up. Nobody listened. I stopped moving, stopped asking. I just closed my eyes and waited for it to end’ (Participant 12)
	Emotional Isolation and Perceived Neglect	«I felt so alone»; «I started to cry»; «No one saw me»; «They gave me medicine but not water»	Profound loneliness accompanied by tearfulness, withdrawal from social interaction, and perception of being overlooked by the healthcare team	‘I felt so alone, even though there were people around me. The nurses were busy, but I needed someone to see me, to really see me. I just cried and cried’ (Participant 17)

**FIGURE 1:**
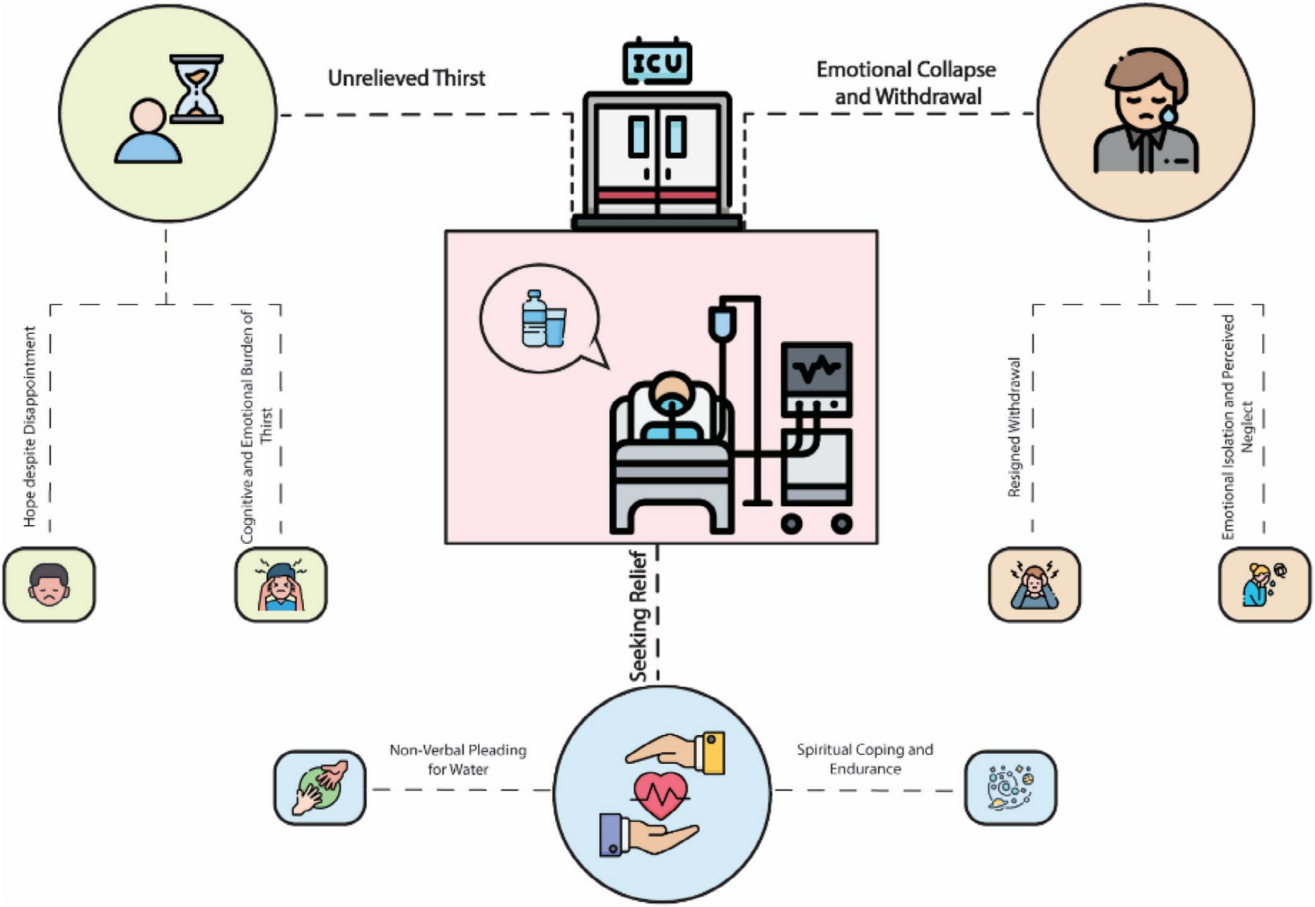
The experience of thirst in ICU patients.

### 3.1 Unrelieved Thirst

The first main category was ‘Unrelieved Thirst’, comprising two subcategories: ‘Hope despite Disappointment’ and ‘Cognitive and Emotional Burden of Thirst’. Participants elaborated on their thirst experience as ongoing and unrelieved, marked by alternating hope and disappointment. They experienced a sense of being caught between hope for relief and constant disappointment, frequently feeling as if they stood on the brink of an abyss or feared sudden death.

#### 3.1.1 Hope Despite Disappointment

Patients were afraid they might die of thirst, which increased their disappointment. Nevertheless, they attempted to console themselves and hoped the drinking restriction would be lifted, allowing someone to eventually give them a drink:

At times, I felt so lost and disconnected, believing no one could truly help. But underlying that despair was a persistent belief that suffering is temporary, and that eventually, I would be helped to overcome this thirst. This deep‐seated hope, even if faint, was what kept me going. (Participant 10)

I kept telling myself that this cannot last forever. One day, someone will understand and give me water. That thought was the only thing that stopped me from giving up completely. (Participant 6)

I told myself that God would not leave me alone. I just needed to be patient a little longer. I believed that someone would eventually come and help me. (Participant 13)

This subcategory illustrates the contradictory presence of hope and despair, where patients felt both close to death and hopeful for rescue. Patients did not passively wait; they actively searched for reasons to believe their thirst would be relieved. Nevertheless, this hope was delicate and could be quickly broken by ongoing disappointments, resulting in emotional fatigue.

#### 3.1.2 Cognitive and Emotional Burden of Thirst

According to participants, severe thirst extended beyond a mere physical sensation, persistently occupying their minds and centering their mental attention on water, relief, and survival. This state resulted in repetitive and intrusive thoughts, leaving patients mentally preoccupied with securing even a minimal amount of water. Simultaneously, patients perceived patience not as a voluntary decision, but as a forced endurance influenced by intense thirst, medical limitations, and doubt about when relief would come:

You keep thinking that you are close to death, and at the same time hoping that someone will come and give you even a drop of water. You want water so badly that you feel like you are about to die. (Participant 3)

Water was all I could think about. Every second, I imagined drinking a glass of cold water. It was like my mind was stuck in a loop, and I couldn't get out of it. (Participant 14)

I told myself maybe this thirst is good for me, maybe it is part of my treatment. But deep down, I knew it was just a way to stop myself from going crazy. (Participant 9)

A fatal feeling overwhelms you, and there is no end. You do not know whether to be patient or not because nothing is clear until you die. I do not know if I will wake up, so I was even afraid to sleep. This pain suffocates you. (Participant 5)

This subcategory encompasses both the cognitive and emotional aspects of unrelieved thirst. Cognitively, patients had intrusive and repetitive thoughts about water that overwhelmed their minds. Emotionally, they suffered through a forced state of patience characterized by fear, uncertainty, and a persistent feeling of suffocation. What makes this subcategory unique is the inclusion of a deliberate coping method known as cognitive reframing. Patients engaged in an active psychological strategy to endure deprivation by framing it as therapeutically necessary, which helped them preserve meaning and resilience instead of passively accepting their situation.

### 3.2 Seeking Relief

‘Seeking Relief’ was the second main category, comprising two subcategories: ‘Spiritual Coping and Endurance’ and ‘Non‐Verbal Pleading for Water’. Patients who could not speak because of intubation sought relief from thirst via two connected but separate routes. One route was spiritual help‐seeking, where patients continuously reached out to God, expressing their need for relief through prayer and inner supplication. The second pathway involved non‐verbal pleading for relief, where patients tried to express their thirst to the treatment team using body language, eye movements, facial expressions, and hand gestures.

#### 3.2.1 Spiritual Coping and Endurance

Participants specifically mentioned relying on prayer and religious stories for strength. For Muslim patients in this study, faith offered comfort and a way to make sense of their suffering. Many found strength in the story of Ashura—the historical event where Imam Hussein (AS) and his companions faced severe thirst before their martyrdom. According to the researchers, these accounts reflect a meaning‐making process that transformed patients' thirst from physical distress into a spiritually significant experience. This conclusion is deeply rooted in Iran's Shia Muslim culture, where the tale of Imam Hussein's thirst in Karbala holds great spiritual significance. Given the potential limitations in transferring this finding to non‐Muslim or non‐Shia communities, future research should explore the presence of analogous spiritual coping mechanisms across diverse cultural and religious contexts:

I always asked God for help. I begged him to give me patience through the six‐month‐old child of Imam Hussein (AS) or to let the nurses give me some water. (Participant 11).

Every night, I prayed to God and asked Imam Hussein to give me strength. I remembered how thirsty he was in Karbala, and I felt closer to him in my suffering. (Participant 16).

I felt that God was with me. I asked him to help me endure this thirst. I knew that if I was patient, I would be rewarded. (Participant 7)

This subcategory focuses on the profound human desire for meaning and spiritual resilience, beyond the practical aim of securing water. Prayer and supplication were not just rituals but active efforts to engage with the divine. This comprehensive resource addressed both physical and emotional needs, making it an exceptionally effective coping strategy.

#### 3.2.2 Non‐Verbal Pleading for Water

This subcategory differs from the prior one, which dealt with the spiritual dimension of coping, by emphasizing the practical, urgent, and physical attempts to communicate thirst to the healthcare team for the immediate purpose of securing water. Due to intubation preventing verbal communication, patients turned to non‐verbal techniques such as tapping the bed rails, hand movements, or expressive facial gestures to engage clinicians:

I have always tried to tell the treatment team that I am thirsty, and they will give me some water. I begged with my eyebrows and eyes for water. You only think of drinking water at that moment, even if you are begging to quench this thirst a little bit. (Participant 8).

I tried so hard to show them I needed water. I moved my hands, I pointed to my mouth, I made eye contact and begged with my eyes. But they didn't seem to understand. (Participant 4)

I kept tapping on the bed to get their attention. I pointed to my mouth and tried to say ‘water’ with my eyes. Sometimes they understood, sometimes they didn't. (Participant 15)

This subcategory reflects the urgent and frequently frustrating efforts to communicate the need for water using gestures, facial expressions, and body language. These actions were not mere casual communications; they were perceived as acts of desperation. The success of these communications relied greatly on the healthcare team's attentiveness, and numerous patients felt disappointed when their gestures were misunderstood or disregarded.

### 3.3 Emotional Collapse and Withdrawal

The third primary category was ‘Emotional Collapse and Withdrawal’, comprising two subcategories: ‘Resigned Withdrawal’ and ‘Emotional Isolation and Perceived Neglect’. Experiencing overwhelming thirst in the ICU environment pushed participants into profound emotional isolation, as they felt trapped by physical weakness and the belief that others did not see their suffering.

#### 3.3.1 Resigned Withdrawal

At first, participants consistently and actively tried to satisfy their thirst, but as their requests for help were repeatedly overlooked or misunderstood, they began to feel increasingly isolated. This ongoing pattern of failure ultimately caused profound psychological and physical fatigue. Eventually, overwhelmed by the futility of their circumstances, the participants gave up seeking help and withdrew into a state of resigned despair:

You do not know what it is like when you cannot ask someone for water, and you are very thirsty. First, you get depressed and tired because you realize that no one understands you, so you stop trying anything else. You want to end this nightmare and die as soon as possible. I give up trying elsewhere. (Participant 1)

After so many tries, I just gave up. Nobody listened. I stopped moving, stopped asking. I just closed my eyes and waited for it to end. (Participant 12)

I stopped asking for water because I knew no one would bring it. I just accepted that I had to suffer. It was easier than hoping and being disappointed again. (Participant 20)

This subcategory explains the psychological shift from actively seeking help to a state of resigned withdrawal. This withdrawal did not indicate acceptance or peace, but instead reflected deep hopelessness. Some participants clearly expressed a desire to die or give up trying (e.g., Participant 1: ‘You want to end this nightmare and die as soon as possible’). According to the researchers, these accounts suggest a profound hopelessness and withdrawal from seeking help actively; nevertheless, it is essential to consider that these interpretations stem from participants' explicit comments and should be evaluated within the framework of their overall ICU experience.

#### 3.3.2 Emotional Isolation and Perceived Neglect

Participants expressed deep loneliness paired with tearfulness and withdrawal, along with a painful sense of being ignored in the ICU. During these times, thirst was felt not just as a physical need but as a frightening crisis worsened by the belief that no one recognized their most fundamental requirement:

Many times, a great sadness came over my heart. I felt very sick and I felt that I was alone. That's why I started to cry. (Participant 18)

I felt so alone, even though there were people around me. The nurses were busy, but I needed someone to see me, to really see me. I just cried and cried. (Participant 17)

I remember once upon a time I was very thirsty. I would die. No one paid attention to the bedside fence. I was suffocating. I was terrified. When my heart rate rises, and I start to sweat, the nurse looked at me and told everyone to run up and start giving me medicine, but it was okay to give me water, but no one understands this. (Participant 19)

This subcategory illustrates the profound emotional isolation and perceived neglect patients experienced during unrelieved thirst. Patients expressed a sense of total aloneness, even when others were present. This loneliness was existential rather than merely physical, embodying the belief that no one fully comprehended their distress. Some participants felt that their physical signs of distress were treated medically instead of being seen as signals of thirst. For instance, Participant 19 expressed that his tachycardia and sweating were managed with medication, while his need for water was overlooked. These accounts suggest that clinicians should consider thirst as a possible contributing factor when these signs are observed, but this finding demands further confirmation through quantitative or interventional studies. This subcategory draws attention to the intense vulnerability of intubated patients who are deprived of their voice in their care.

## 4 Discussion

This study investigated ICU patients' experiences of thirst after extubation and their coping mechanisms, identifying three primary categories: ‘Unrelieved Thirst’, ‘Seeking Relief’, and ‘Emotional Collapse and Withdrawal’. In the first category, Unrelieved Thirst, patients reported experiencing endless waiting and intrusive thoughts about water that worsened their suffering, with poor communication adding to their distress. This unrelieved thirst led patients to the second category, Seeking Relief, where they resorted to spiritual coping and non‐verbal requests for water to ease their discomfort. If these needs went unmet, patients progressed to the third phase, Emotional Collapse and Withdrawal, where they emotionally withdrew and experienced feelings of isolation and neglect. Our findings are consistent with the Theory of Unpleasant Symptom, which frames thirst as multidimensional (Lenz et al. 1997). In this study, fluid restriction and intubation, alongside anxiety, hopelessness, impaired communication, and the ICU environment, produced an intense symptom experience. Additionally, the findings were consistent with Hosseini et al. (2020), who observed frustration when expectations for caregiver interaction were not met (Hosseini et al. 2020). However, our study emphasizes how intubation‐induced inability to express thirst exacerbated frustration and altered time perception, as thirst is an urgent physiological need distinct from general communication difficulties, becoming psychologically overwhelming when patients are voiceless and dependent; thus, interventions focusing solely on communication may not suffice, and tackling the unmet need itself is equally vital.

In line with Guttormson et al. (2015) and Holm and Dreyer (2017), intubated patients experienced persistent and frustrating thirst, as the endotracheal tube and ICU limitations hindered meeting basic needs and expressing them, leading to ongoing thirst and feelings of deprivation, disappointment, and helplessness (Guttormson et al. 2015; Holm and Dreyer 2017). The current investigation showed that thirst threatened patients' sense of self and agency, with the study's novelty being its exploration of thirst as a multidimensional experience that goes beyond physical sensation to include cognitive, emotional, spiritual, and existential aspects. The progression from hope to cognitive burden, help‐seeking, and emotional collapse is uniquely highlighted in this study; moreover, thirst‐induced distress might be wrongly seen as physiological instability, revealing a gap in clinical recognition.

The temporal distortion found in unrelieved thirst corresponds with Minton et al. (2018), who showed that waiting affected time perception because of sensory monotony and lack of circadian markers. In contrast, Granberg et al. (1999) observed a similar disruption in kidney patients, attributing the distortion to accumulated fatigue and prolonged dialysis (Granberg et al. 1999). Likewise, Hajiabadi et al. (2017) found that ventilated patients faced distress due to waiting and unmet emotional needs (Hajiabadi et al. 2017), which aligns with our subcategories of ‘Hope Despite Disappointment’, ‘Cognitive and Emotional Burden of Thirst’, and ‘Stressful Patience’. These similarities indicate that although temporal distortion seems alike across groups, its root causes vary: environmentally influenced in the ICU, linked to chronic illness in kidney care, and stemming from unmet needs during extended waiting in ventilated patients.

Seeking Relief in our study was reflected in two subcategories: ‘Spiritual Coping and Endurance’ and ‘Non‐Verbal Pleading for Water’. When patients could not self‐manage thirst or communicate their needs, they relied on spiritual and non‐verbal strategies to seek help. Spirituality served as a source of meaning and coping, aiding patients in enduring suffering and sustaining hope despite physical limitations and dependence. Concerning ‘Non‐Verbal Pleading for Water’, Duffy et al. (2018) found that ventilated patients in surgical ICUs expressed wider needs by reaching out to nurses, family, or clergy, with the nature and intensity showing significant differences despite aligning with this theme (Duffy et al. 2018). In our study, patients were unable to communicate verbally, and their help‐seeking involved desperate non‐verbal signals—such as gaze and bodily gestures—specifically indicating intense thirst. This discrepancy may stem from our emphasis on thirst as a singular, urgent need, while Duffy et al. investigated a wider variety of needs. In the Iranian context, cultural influences could have impacted the degree of non‐verbal pleading, with direct expressions of thirst possibly viewed as burdensome. These subcategories pointed to two related dimensions of coping—seeking support from transcendent sources and from other individuals—underscoring the significance of meeting both spiritual and interpersonal needs in ICU care. Quinn et al. (2023) found that hospitalized patients and healthcare workers regularly had ‘sacred moments’—brief but significant experiences of human and spiritual connection—associated with comfort, meaning, lowered stress, and improved patient–provider relationships (Quinn et al. 2023). Satisfying spiritual and non‐spiritual needs in the ICU inspired hope and reduced distress.

The category ‘Emotional Collapse and Withdrawal’ illustrated the significant emotional burden from unrelieved thirst, mainly when patients lacked effective communication and depended on others for relief. Its subcategories, including ‘Resigned Withdrawal’ and ‘Emotional Isolation and Perceived Neglect’, captured different but linked dimensions of this suffering. Whereas Tate et al. (2012) reported anxiety, suffocation, and fear of death in mechanically ventilated patients—commonly associated with coughing and ventilator dis‐synchrony (Tate et al. 2012), the present study results indicated participants interpreted similar symptoms as possibly stemming from unacknowledged severe thirst.

Our qualitative data do not establish a cause‐and‐effect relationship between thirst and physiological signs such as tachycardia; rather, participants identified thirst as the main source of their distress, a perception that was overlooked by the healthcare team. The case of Participant 19 was especially revealing: tachycardia and significant distress were treated with medication, even though the patient clearly identified thirst as the underlying issue. Beyond clinical misunderstanding, this case exposed a failure in human connection, with suffering addressed medically instead of being understood through a relational approach. Holopainen et al. (2014) supported this idea by demonstrating that patients may be isolated with their fears if human relationships are absent (Holopainen et al. 2014). This contrast highlights how clinical misinterpretation of thirst‐related distress can result in pharmacological treatment while the root cause is ignored, with unrelieved thirst worsening psychological suffering and risking concealment through symptom medicalization. Kjeldsen et al. (2018) also discovered that ICU patients linked thirst with feelings of hopelessness, anxiety, and helplessness, highlighting that thirst goes beyond physical sensation to become a profound psychological experience (Kjeldsen et al. 2018). In general, these studies indicate that thirst in the ICU is not only distressing but often goes unrecognized when patients are unable to communicate, with their suffering mistaken for physiological decline instead of an unmet basic need.

### 4.1 Implications for Nursing Practice

This qualitative study's findings indicate several possible implications for nursing practice. First, routine visual assessment of thirst in intubated patients could be considered since these patients might not be able to verbally express this need. However, further evaluation is needed to determine the feasibility, acceptability, and impact of such assessments. Second, because nurses often misunderstood non‐verbal cues such as hand gestures and facial expressions, exploring training for them to better recognize and respond to these subtle signals might be a valuable practice consideration, but its effectiveness should be evaluated in future studies. Third, recognizing and supporting spiritual coping resources, when suitable, could be a helpful approach, as these resources seemed to provide significant endurance for some patients, though further evaluation is needed. Fourth, the tendency to medicalize thirst‐induced distress (like tachycardia being diagnosed as anxiety) points to a need for heightened clinical attention to thirst as a potential cause. Nevertheless, such physiological indicators should be regarded as patient‐experienced signs connected to thirst instead of physiological effects demonstrated by the current study. Ultimately, even if oral intake is impossible, recognizing the patient's request and offering timely comfort‐focused mouth care might alleviate feelings of helplessness, shame, and isolation reported by participants, although the efficacy of this method is yet to be confirmed. These implications should primarily be considered as generating hypotheses and calling for additional study, rather than as recommendations grounded in evidence.

### 4.2 Study Limitations

The study faced several limitations, mainly its dependence on retrospective reports of thirst during mechanical ventilation, as interviews done after extubation could have been affected by sedation, critical illness, delirium, altered consciousness, pain, fatigue, and cognitive changes following extubation, although CAM‐ICU was used to exclude patients with delirium at the interview, episodes of transient delirium or altered consciousness during the ICU stay may have affected memory formation and recall. Furthermore, interviews occurred within hours after extubation, though some patients suffered from hoarseness or tube‐related pain requiring breaks or rescheduling, and the busy, noisy ICU setting interrupted interviews despite collaboration with staff. Furthermore, the interviewer, a female nursing PhD and faculty member, might have affected responses, and despite encouraging participants to speak openly, social desirability bias cannot be ruled out; primary coding by one researcher may have caused bias, although codes and categories were examined with co‐authors. In addition, clinical heterogeneity could limit how well findings apply to specific groups, since despite diverse diagnoses offering wide insight, thirst may be experienced differently by patients with particular conditions (e.g., cancer, liver failure). Moreover, only patients who were relatively alert (RASS −1 to +1, CAM‐ICU negative) and able to communicate were selected, excluding those with reduced consciousness, severe delirium, or communication impairments; given the prevalence of delirium in ICU patients and its possible link to thirst, future research should focus on this population. Finally, given that spiritual coping strategies—especially those involving Ashura and Imam Hussein—are deeply embedded in Iran's Shia Muslim culture, their transferability to non‐Muslim or non‐Shia communities could be limited; furthermore, as the study was carried out in three hospitals in Tehran, the outcomes may not be applicable to contexts with differing cultural, organizational, or resource factors.

## 5 Conclusion

This study reveals that thirst in mechanically ventilated ICU patients is more than just a physical symptom; rather, it is a deep, multidimensional experience that worsens suffering when patients are unable to express their needs. When thirst is ignored or misunderstood, it can become a neglected cause of physical, emotional, and existential distress. These findings therefore emphasize the necessity for increased clinical focus on thirst as a basic human need and for prompt, compassionate care. In addition, our results indicate that communication barriers caused by intubation can render thirst ‘invisible’, so depending on patient‐initiated requests might not be enough. Participants perceived their thirst‐related distress as being interpreted as anxiety, agitation, or physiological instability, indicating that clinicians should consider thirst as a potential underlying cause when these symptoms arise; however, this observation is based on participant perception and requires additional research. Moreover, acknowledging the request and promptly providing mouth care can lessen feelings of helplessness, shame, and isolation even when oral intake isn't possible. Consequently, future interventional and quantitative research is necessary to assess routine thirst evaluation with visual scales in ICU protocols and clinician training.

## Author Contributions

**Alireza Sadeghi:** conceptualization, data curation, formal analysis, methodology, project administration, visualization, writing – original draft, writing – review and editing. **Amir Mohamad Nazari:** methodology, project administration, visualization, writing – original draft, writing – review and editing. **Alun C. Jackson:** methodology, project administration, visualization, writing – original draft, writing – review and editing. **Elham Khedmati:** data curation, methodology, project administration, visualization, writing – original draft, writing – review and editing. **Fatemeh Bahramnezhad:** conceptualization, formal analysis, methodology, project administration, visualization, writing – original draft, writing – review and editing.

## Funding

The authors have nothing to report.

## Disclosure

Fatemeh Bahramnezhad, the corresponding author, confirms that this manuscript truthfully and accurately represents the study carried out, with no essential parts of the research left out and all deviations from the original or registered study design fully clarified.

Artificial intelligence (AI) usage: This study did not employ artificial intelligence (AI) tools to translate participant quotations or raw interview data; AI‐assisted language editing was solely used to enhance the clarity, grammar, and style of the English manuscript post‐translation, with all AI‐generated edits reviewed, verified, and approved by the authors. No identifiable participant information or raw interview content was input into any AI tool, and participant confidentiality was preserved during the entire study.

## Ethics Statement

Adhering to the Helsinki Declaration's ethical standards, this study was approved by the Ethics Committee of Tehran University of Medical Sciences (No. IR.TUMS.MEDICINE.REC.1400.405). Participants were made aware of the study's purpose, assured of privacy and confidentiality, and informed that participation was voluntary with the option to exit whenever they wished. Additionally, participants completed the informed consent forms prior to taking part in the study.

## Consent

Before enrollment, written informed consent was secured from all participants or, when relevant, from their legally authorized representatives.

## Conflicts of Interest

The authors declare no conflicts of interest.

## Data Availability

The data supporting this study's findings are available from the corresponding author upon request.
